# *Vibrio cholerae* in Traveler from Haiti to Canada

**DOI:** 10.3201/eid1706.110161

**Published:** 2011-06

**Authors:** Matthew W. Gilmour, Valérie Martel-Laferrière, Simon Lévesque, Christiane Gaudreau, Sadjia Bekal, Céline Nadon, Anne-Marie Bourgault

**Affiliations:** Author affiliations: Public Health Agency of Canada, Winnipeg, Manitoba, Canada (M.W. Gilmour, C. Nadon);; Centre Hospitalier de l’Université de Montréal, Montreal, Quebec, Canada (V. Martel-Laferrière, C. Gaudreau, A.-M. Bourgault);; Institut National de Santé Publique du Québec, Ste-Anne-de-Bellevue, Quebec (S. Lévesque, S. Bekal, A.-M. Bourgault)

**Keywords:** cholera, Vibrio cholerae O1 Ogawa, bacteria, outbreak, Haiti, Canada, traveler, expedited, letter

**To the Editor:** A nationwide outbreak of cholera caused by *Vibrio cholerae* O1 serotype Ogawa began in Haiti in October 2010 and has since resulted in >200,000 illnesses and 4,000 deaths (*1*). Additional cases of cholera attributed to the outbreak strain have subsequently been reported in the neighboring Dominican Republic and in Florida and New Jersey in the United States. In these instances, illness was related to travel to Haiti or consumption of contaminated water on the island of Hispaniola (which is shared by Haiti and Dominican Republic).

In Canada, the province of Québec has a large Haitian immigrant population. In early November 2010, the Québec public health authorities provided clinicians and laboratories with recommendations regarding the diagnosis of *V. cholerae* infections. We report a case of *V. cholerae* O1 serotype Ogawa in Canada related to the outbreak in Haiti. It was diagnosed in Montréal, Québec, on January 5, 2011.

A 49-year-old Canadian woman traveled to Haiti with her 5 brothers and sisters during December 22–29, 2010, to attend her mother’s funeral. While in Haiti, they stayed with family members. She came to the emergency department of the Centre Hospitalier de l’Université de Montréal on January 1, 2011, with abdominal cramps and diarrhea of moderate intensity that had started on December 29, the day she returned from Haiti. The patient was asthenic, but vital signs and results of a physical examination were normal. A complete blood count, levels of serum electrolytes and serum creatinine, and results of liver function tests were within reference ranges. A fecal sample was submitted and the patient received intravenous fluids and 1 dose (300 mg) of doxycycline. She improved rapidly and was discharged on January 3. The patient returned to the outpatient clinic on January 7, and she had recovered from her illness. Control fecal specimens obtained on January 9 and 10 were negative for *V. cholerae*. Family members that traveled with her did not get ill, and there were no secondary cases among her family members in Montréal.

The fecal culture of the sample provided on December 29 contained *V. cholerae.* The isolate was confirmed as toxigenic *V. cholerae* serogroup O1, serotype Ogawa, biotype El Tor, and matched the Haiti outbreak strain when tested by pulsed-field gel electrophoresis (PFGE). Antimicrobial drug susceptibility testing was performed by continuous gradient dilution (Etest; AB Biodisk, Solna, Sweden), and results were interpreted according to standard criteria (*2*). The strain was susceptible to azithromycin (0.25 mg/L), ciprofloxacin (0.5 mg/L), and tetracycline (1 mg/L) and resistant to trimethoprim/sulfamethoxazole (>32/608 mg/L).

Cases of diagnosed cholera are rare in Canada (0–3 laboratory-confirmed isolations of serogroup O1 and O139 *V. cholerae* per year (*3*). All cases in Canada have been associated with travel to cholera-endemic areas, including Africa and Southeast Asia. Monitoring of cholera in Canada is completed through the National Notifiable Diseases Program and through the public health laboratory network. Biochemical identification, serotyping, and PFGE testing are performed on all suspected *V. cholerae* isolates. Confirmed isolations of a serogroup O1 or O139 *V. cholerae* strain that produces cholera toxin are also reported through the International Health Regulations focal point.

Whole genome sequencing has been completed for several isolates to investigate the origin of the Haiti cholera outbreak (*4**,**5*). However, PFGE remains one of the primary tools for defining the outbreak strain (*4*). The highly standardized methods of PulseNet International for generating, analyzing, and comparing PFGE patterns are used worldwide to track the temporal and geographic distribution of *V. cholerae* (*6**,**7*).

PFGE for *V. cholerae* was performed by using restriction enzymes *Sfi*I and *Not*I (*8*). The PFGE patterns of this travel-associated case matched patterns of the representative Haiti cholera outbreak strain that was deposited into the American Type Culture Collection (Manassas, VA, USA) by the Centers for Disease Control and Prevention (Atlanta, GA, USA; strain BAA-2163; CDC isolate 2010EL-1786) (Figure). PulseNet Canada *Sfi*I and *Not*I PFGE pattern designations were VCSFI.0006 and VCNTI.0006, respectively, and were equivalent to PulseNet USA patterns KZGS12.0088 and KZGN11.0092.

**Figure:**
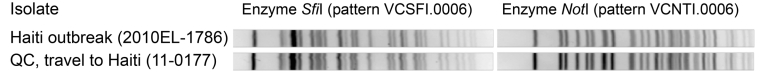
Pulsed-field gel electrophoresis (PFGE) of cholera outbreak strain from Haiti and travel-associated isolate in the patient, by using *Sfi*I and *Not*I and PulseNet Canada PFGE pattern designations.

Prevention, treatment, and control efforts are currently under way in Haiti. Loss of infrastructure during the earthquake of January 12, 2010, has affected implementation of sanitation and public health measures. Travel advisories and travel health precautions were subsequently released, including those from Canada and United States (*9**,**10*). These precautions recommended that preventative measures such as vaccination and safe food and water consumption practices be adhered to by residents and visitors to affected regions. Although the public health community anticipated that travel-associated cases would be diagnosed in Québec, this report of a documented case (supported by laboratory and epidemiologic data) emphasizes the domestic and international public health risk caused by the nationwide outbreak in Haiti. It also illustrates the need for an accurate travel history in clinical and laboratory diagnosis of cholera infections.

## References

[1] Pan American Health Organization. PAHO responds to cholera outbreak on the island of Hispaniola; 2011 [cited 2011 Mar 28]. http://new.paho.org/disasters/index.php?option=com_content&task=view&id=1423&Itemid=1

[2] Clinical and Laboratory Standards Institute (CLSI). Methods for antimicrobial dilution and disk susceptibility testing of infrequently isolated or fastidious bacteria. M45A2. Wayne (PA): The Institute; 2006. 10.1086/510431 17173232

[3] Public Health Agency of Canada. Laboratory surveillance data for enteric pathogens in Canada. Annual summary 2006 [cited 2011 Mar 28]. http://www.nml-lnm.gc.ca/NESP-PNSME/assets/pdf/2006AnnualReport.pdf

[4] Centers for Disease Contro land Prevention. Update: outbreak of cholera—Haiti, 2010. MMWR Morb Mortal Wkly Rep. 2010;59:1473–9.21085088

[5] Chin CS, Sorenson J, Harris JB, Robins WP, Charles RC, Jean-Charles RR, The origin of the Haitian cholera outbreak strain. N Engl J Med. 2011;364:33–42. 10.1056/NEJMoa1012928 21142692 PMC3030187

[6] Swaminathan B, Gerner-Smidt P, Ng LK, Lukinmaa S, Kam KM, Rolando S, Building PulseNet International: an interconnected system of laboratory networks to facilitate timely public health recognition and response to foodborne disease outbreaks and emerging foodborne diseases. Foodborne Pathog Dis. 2006;3:36–50. 10.1089/fpd.2006.3.36 16602978

[7] Staley C, Harwood VJ. The use of genetic typing methods to discriminate among strains of *Vibrio cholerae, V. parahaemolyticus*, and *V. vulnificus.* J AOAC Int. 2010;93:1553–69. 21140669

[8] Cooper KL, Luey CK, Bird M, Terajima J, Nair GB, Kam KM, Development and validation of a PulseNet standardized pulsed-field gel electrophoresis protocol for subtyping of *Vibrio cholerae.* Foodborne Pathog Dis. 2006;3:51–8. 10.1089/fpd.2006.3.51 16602979

[9] Update on travel to Haiti: cholera outbreak [cited 2011 Mar 28]. http://www.phac-aspc.gc.ca/tmp-pmv/thn-csv/quake-tremble-haiti-eng.php

[10] Centers for Disease Control and Prevention. Cholera in Haiti: CDC travelers’ health [cited 2011 Mar 28]. http://wwwnc.cdc.gov/travel/content/travel-health-precaution/haiti-cholera.aspx

